# Comparative Performance Evaluation of Multiconfiguration Touch-Trigger Probes for Closed-Loop Machining of Large Jet Engine Cases

**DOI:** 10.3390/ma15041461

**Published:** 2022-02-16

**Authors:** Grzegorz Szyszka, Jarosław Sęp

**Affiliations:** 1 Department of Manufacturing Processes and Production Engineering, Ignacy Łukasiewicz Rzeszów University of Technology, ul. Powstańców Warszawy 12, 35-959 Rzeszów, Poland; jsztmiop@prz.edu.pl; 2 Pratt & Whitney Rzeszów S.A., ul. Hetmańska 120, 35-078 Rzeszów, Poland

**Keywords:** stainless steel, thin-wall machining, machine tool, on-machine measurement, touch-trigger probe, part probing

## Abstract

This article presents advances in the methodology of rapid various probe configurations comparison for the five-axis, tilting-head machine tools in conjunction with master artifacts. The research was performed in a direct context of automated machining of large, complex jet engine cases made from 17-4PH and 321 stainless steel materials. The aim of the study was to investigate whether all probe configurations have comparable measurement capability for use in manufacturing environment conditions. Based on the preliminary stage of the study, the T1 main straight probe achieved acceptable results of repeatability and reproducibility, lower than 10%, except for the reference diameter measurement of MT#2, where 15.4% R&R was achieved, conditionally accepted. For the straight probe configuration, error lower than 10 μm was achieved for the true position measurement and error ±10 μm for the reference diameter measurement, in relation to the vertical and horizontal head position, with the exception of the T9 and T5 MT#2 probe configuration, where higher error was noticed. The obtained results of the T5 MT#2 and T9 probes were supplemented with additional tests, which are also included. For the custom styli probes, the T4 and T6 configurations, unacceptable error, higher than 0.30 mm, was observed for the *Y* axis position. Depending on the shop floor and machine tool condition, variability of the results was also observed. Hence, the collected data and research helped to determine the mutual measurement errors and determine the application limitations of probes for an adaptive process flow.

## 1. Introduction

Nowadays, the design of aerospace jet engines requires the usage of advanced materials with high mechanical demands, high corrosion resistance, abrasion resistance, and high hardness, in various temperature conditions [1,2]. The specific requirements are fulfilled by hard-to-cut materials including titanium, cobalt and nickel alloys, and specific grades of stainless steel [1,3].

Stainless steel, thanks to the material properties, is widely used in applications for jet engine part manufacturing.

The martensitic 17-4PH stainless steel (e.g., AMS 5643) and austenitic 321 stainless steel (e.g., AMS5645) are applicable for engine cold section components such as engine casing (from small to large >ø1000 mm parts) due to their good mechanical properties, corrosion resistance, heat resistance, comparatively good manufacturing ability of fabrication, welding, and machining, and acceptable cost [3,4,5].

The martensitic, solution heat-treated, precipitation-hardened 17-4PH stainless steel contains 15–17.5% chromium, 3–5% nickel, and 3–5% copper, along with manganese, silicon, niobium, and molybdenum [5,6]. According to the SAE AMS 5643 standard, the material has a tensile strength of 931–1310 MPa and a yield strength of 105–170 MPa. The machining ability range is determined up to the 30–40% level and may vary according to the material condition and hardness of the metal (277–444 HB) [6].

The austenitic, solution heat-treated 321 stainless steel contains 17–19% chromium, 8–12% nickel, and up to 2% of manganese, along with titanium, molybdenum, silicon, copper, and nitrogen [7]. According to the SAE AMS 5645 standard, the material has a tensile strength of 517 MPa and a yield strength of 207 MPa. The machining ability range is determined up to the 35–45% level and may vary according to the material condition and hardness of the metal (up to 255 HB) [7].

Due to the relatively high density of 7.75–7.92 g/cm^3^ (compared to other materials used in the cold section of engines), the stainless steel structural parts are designed as thin-walled components in order to reduce the weight and manufacturing cost. Additionally, the large sheet metal engine cases are characterized high part complexity and integrated functionality.

**Figure 1:**
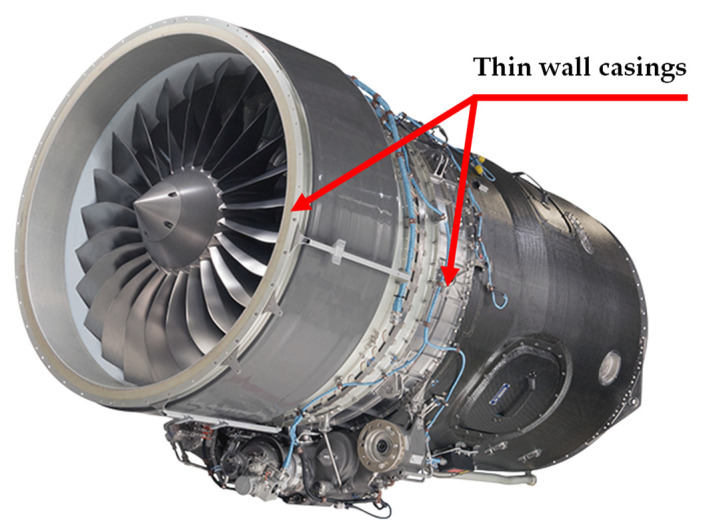
Example of new-generation jet engine with complex cases made from various grades of stainless steel, 17-4PH and 321 [8].

Figure 1 shows an example of a large jet engine case made from various grades of stainless steel materials.

The difficulty of machining of selected stainless steel grades results from their high tensile strength, high ductility, high work-hardening rate, low thermal conductivity, and abrasive character [2]. The machining, due to the material properties, generates relatively high heat emission, high cutting forces, difficulties with chip braking, built-up edge formation, and a high tool wear rate [2,9]. These phenomena reduce the machinability and directly affect parts’ behavior during machining in relation to the surface finish, dimensional accuracy and dimensional stability, and tool life [2,5,10,11].

The machining of thin-wall, complex casings is usually challenging and difficult due to the relatively large component size, frequent and self-excited part vibrations, parts’ susceptibility to deflection and tool chatter due to limited rigidity resulting from long overhang, and slim tool design for access to hard-to-reach areas [3].

The next issue of the machining of large stainless steel cases results from the necessity of multiple, stepped tool passes with a limited depth of cut and limited cutting speed and feed on all machining stages, including roughing, semi-finishing, and finishing, to reduce the risk of part deflection and to achieve requirements of dimensional and geometrical tolerances of multiple machined features [12].

The manufacturing processes of jet engine parts has to be prepared to ensure maximum quality and production performance and the lowest manufacturing cost [9]. The machining concept has to be considered to take control of the numerous issues and challenges to ensure a stable and robust manufacturing process.

One of the solutions is the application of a closed-loop, adaptive process, performed on modern five-axis multitasking machine tools (mill/turn) with integrated, advanced part probing system capability [12].

Measuring technologies are currently available on modern machine tools and are widely used for efficient manufacturing. The closed-loop process chain is especially notable for its complex part machining, which does not interrupt manufacturing procedures by external measurements [13].

In many cases, the machining process of CNC multitasking machines has minimal human intervention [14]. The significant growth of automated machining processes can also be observed in the aviation industry. The production of advanced components for jet engines is generally characterized by fewer pieces in the production batch, long machining times, machining complexity, high demand for quality parts, process accuracy, and repeatability. This type of production is possible by implementing closed-loop processes with wide touch-trigger probes (TTP) application [15,16,17].

In the aviation industry, five-axis multitasking machine centers enable the implementation of complete machining in one clamping; this is accomplished by combining many types of machining functions, including turning, drilling, milling, boring, tapping, and grinding. These functions help to obtain geometrically complex elements that maintain the subject’s required dimensional and geometrical tolerance [15,16,18,19].

Part probing technology built-in machine tools (MTs) allow significant productivity increases, reduce human error, and develop a robust manufacturing process. 

Part probing technology is currently used for workpiece setups and pre- and post-process part inspection. TTPs are often used for the in-process validation of dimensions, enabling the machine’s response to process variation and creating a closed-loop process adaptation based on dimensional part behavior during processing; this reduces the risk of non-conforming parts, which is important for the machining and significant workpiece cost of aviation parts. Measuring probes have also become essential for MTs condition checkup, kinematics, and geometric error compensation in machine tools. The complexity of TTP applications has led to treating MTs and on-machine measurement (OMM) as holistic machining systems [13,20,21].

The common and optimal usage of on-MT measurements is limited by disadvantages, such as:

- MT measurements should be limited and simplified as much as possible because medium/large MTs are more expensive than CMM, and the CNC machine working time is more valuable than the CMM working time;
- Lack of long-term MT accuracy, which is affected by various errors [22];
- Machine not stable enough due to environmental condition changes and influence of the machining process [23];
- Lack of MT traceability due to both measurement and machining being performed on the same equipment, which may not recognize nonconformance from repeated geometric and dimensional errors [13].

There is a variety of TTP behavior, in the case of the multi-probe system, because each probe is a separate tool, which distinguishes machine tool systems from CMM systems [24]. Determining inaccurate measurements using TTPs is a complex problem due to the influence of different error sources that are not fully understood yet. Many different factors affect probing performance; therefore, probing errors must be considered in assessing MTs for accuracy and repeatability [13].

**Figure 2:**
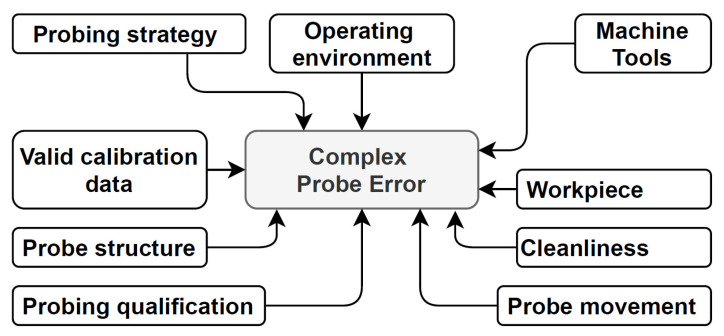
Items for probing error budget of on-machine measurements [13,25,26,27].

The essential factors in total probing error budgets are shown in Figure 2; they are organized into seven categories consisting of several factors. The operating environment comprises the shop floor, the MT workspace temperature, and the temperature’s long-term stability. The environmental variation affects measurements due to changes in both the MT and part behavior. Probe structure includes probe deflection, stylus length and shape, stylus tip type, and tip wear. Probe movement includes feed rate, direction, and force of tip impact, approach strategy (single or double touch), and stylus position (vertical, horizontal, or angled). The workpiece category consists of the type and shape of features and surface condition. The cleanliness category includes the cleanliness of the surface, spindle/tool shank, and stylus tip, which directly affect the measurement results.

The probing strategy consists of measurement paths and the number of points to measure. The machine tools category includes machine geometry and kinematics condition, accuracy and repeatability of positioning, and the spindle probe load’s reproducibility. Valid calibration data describe calibration data errors in reference to the probe and machine tool setup. The last category, probing qualification, comprises measuring procedures to identify probing errors and rules for uncertain measurement levels [13,25,26,27].

The complexity of machine components in the aviation industry demands access to hard-to-reach areas for feature measurements; therefore, it is necessary that custom or special TTPs and styli shapes fulfill the process requirements. 

The implementation of a closed-loop process is possible provided that the probing system can be used for in-process and post-process measurements of parts on the machining level [28]. Hence, it is necessary submit to control determining the variability of a measurement process across the Measuring System Analysis (MSA) statistical methods such as Repeatability and Reproducibility (R&R) [28,29].

Acceptable probing system capability is important due to the fact that MTs and probes are used under variable shop floor conditions and the fact that the measurement results affect the final part dimensions.

Jacniacka et al. [25] studied the inspection probe uncertainty based on the measurement of the coordinates of the point, one- and two- dimensional length measurement, and length measurement using multiple measurement strategies. The achieved short-term results were stable and accurate; however, an impact on the results of machine tool geometry accuracy was observed also on the small CNC machine tool.

Bomba et al. [28] tested a probing system based on the R&R method and statistical process control (SPC) of straight probes while carrying out an evaluation based on continuously collected data. A high R&R level was achieved and the SPC parameters of the analyzed system in relation to the manufacturing application were good.

Sepahi-Boroujeni et al. [30] presented an advanced and complex method of part probing repeatability investigation and prediction in conjunction with a single probe in any probing position of a five-axis, tilting-table machine tool based on a spherical artifact and ring gauge artifact. The general model of probing repeatability was evaluated. It was found that the tested repeatability model can reliably portray the random behavior of a machine tool.

Holub et al. [31] studied the application of a machining center with a touch-trigger probe as a measuring device. The probing system capability was verified based on a combined method with length dimension measurement by the straight touch probe in conjunction with a laser interferometer application. The research found that the achieved probing results were acceptable with tolerance greater 0.015mm. However, the authors recognized that it will be difficult to maintain similar results on large, heavy-duty machining centers on the real shop floor condition.

The ISO 230–10:2016 [27] standard presents guidelines for the evaluation of probing system repeatability based on master gauge measurement and data records of the *X*, *Y*, and *Z* axis in the specific position of the machine tool only [30] and a simple probe configuration solely.

However, none of these articles evaluated measurement quality in conjunction with five-axis, tilting-head machine tools and with various touch-trigger probe configurations.

This article presents an alternative approach, a comparative performance analysis method of multi-configuration of TTP error evaluation. The presented methodology is a combined method with initial R&R study and a rapid method for measurement check ability, new probe implementation, probe correlation, and as a support tool of probing issue investigation. The presented method is not intended to eliminate the MSA methodology of probing systems or quality evaluation with methods of MT error identification [22]; however, it can support statistical methods as a quick and direct shop floor diagnostic tool for probing and MT quality evaluation.

The research of the presented methodology was developed directly for closed-loop machining processes of large, thin-walled jet engine cases made from stainless steel materials with the use of complex probing systems on five-axis, tilting-head, multitasking machines based on manufacturing plant requirements.

The study includes the development of the method, the design and preparation of a dedicated master part, NC tape preparation, execution of tests, data acquisition and handling of initial, main, and support tests, and recognized issue investigation. Section 2 describes the probing system used in regular manufacturing. Section 3 presents details of the purpose of the comparative performance evaluation methodology. Section 4 describes the data collection steps and results discussion. Section 5 presents a summary of the study.

## 2. Probing System Description

New-generation jet engine designs demand higher dimensional and geometrical accuracy for parts. The aviation market also demands higher performance for machining processes. New ideas are needed for closed-loop machining on new generations of MTs [7,12].

One of the key technologies for satisfying these requirements is advanced, customized, or specialized part probing systems developed and implemented for five-axis multitasking machines. The design complexity of parts for new jet engine cases and process requirements for features in process measurement demand various configurations of probe shapes.

**Figure 3:**
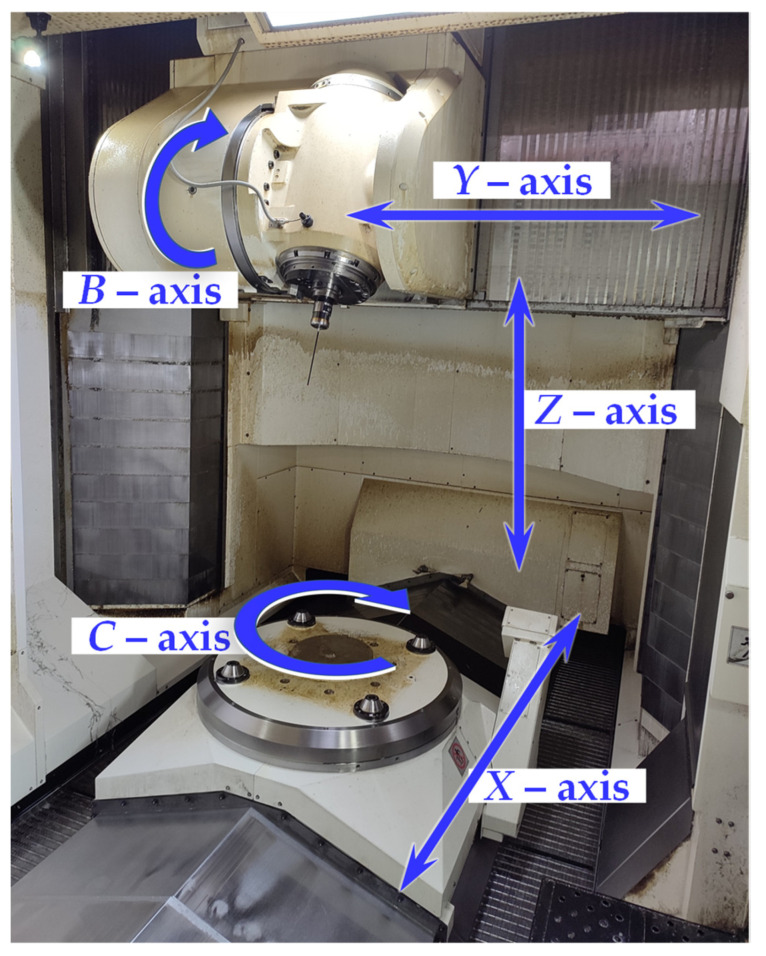
General view of the 5-axis multitasking machine tool used in our study—MT kinematics and axis structure view.

The MT measurement system consists of a five-axis CNC multitasking machining center with a tilting head, a direct measurement system for all linear and rotary axes, five Renishaw (Renishaw plc, United Kingdom, New Mills, Wotton-under-Edge) RMP600 highly accurate strain gauge probes, and three modular RMP60M with LP2 kinematic probes connected to an RMI-Q interface (radio transmission system). The MT and its probing system are located on the shop floor and use the turning and milling mode during regular production. General data for the MT configuration are shown in Table 1 [32] and Figure 3.

**Figure 4:**
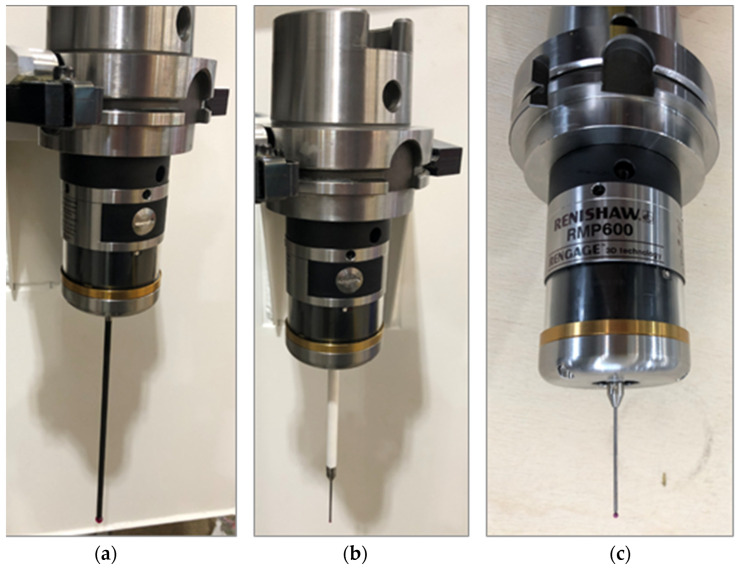
Configuration of RMP600 strain gauge probes with straight stylus shape, various lengths, stylus type, and tip diameter. Detailed data shown in Table 2. (**a**) T1 probe, (**b**) T2 probe, (**c**) T9 probe.

**Figure 5:**
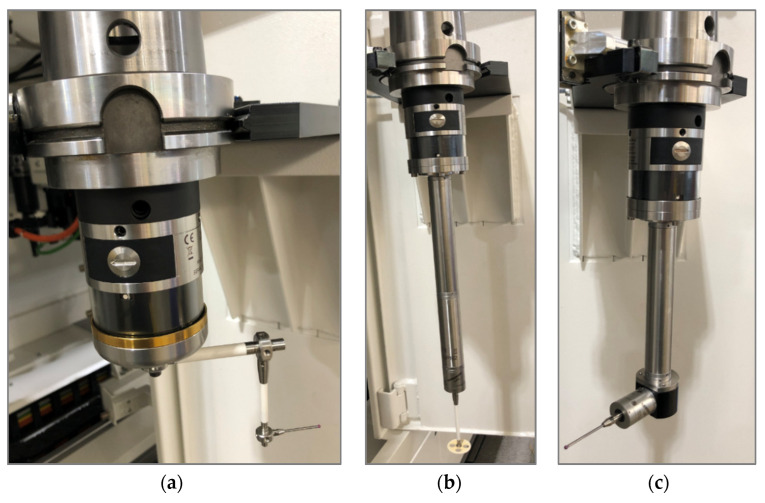
Configuration of TTP: special RMP600 and RMP60M + LP2 strain gauge probe. Detailed data shown in Table 2. (**a**) T4A/B probe, (**b**) T5 probe, (**c**) T6 probe.

In this article, we present only six probes due to IP restrictions. The technical specifications of these probes are described in Table 2. The configuration of TTP shapes is shown in Figure 4 and Figure 5. 

The measurement system consists of the dedicated software Renishaw Inspection Plus Special [33] for all service probes. The software was developed as a special solution with expansion possibilities for future application. Measurement results are written automatically in .txt or .csv files with additional reporting software.

Figure 4 shows probes T1, T2, and T9, which measure features directly with a straight stylus. The tip size, ball diameter, and stylus length depend on the part dimensions and surface shape configuration. Figure 5 shows probes T4A/T4B, T5, and T6, which are used for hard-to-reach feature measurements, such as grooves, internal areas, the surfaces of bottom flanges, and diameters. T4B is the same probe as T4A but rotated at 180° by the spindle index. The T5 probe is used for measuring deep holes. The T1 and T5 probes are also used for machine state inspection, kinematic parameter compensation, and reducing the temperature variability on the shop floor from influencing machine tools.

## 3. Probing Methodology

### 3.1. General Description of the Study

The tests were performed on two MTs (the same type), with a dedicated set of probes, used in regular production under shop floor conditions. The main measurements were performed within one year of MT utilization. The sequence of research was divided into three stages as follows:

- Initial stage, R&R evaluation of main T1 probe, to establish repeatability and reproducibility index value in relation to the machining process tolerances;
- Comparative performance evaluation with preliminary stage as proof of concept—performed for T1, T2, T9 straight probes and MT#3 only;
- Comparative performance evaluation, main measurements, system performance analysis of all types of probes—five tests on MT #3 and six tests on MT #2 performed.

### 3.2. R&R Measurement Assumptions

**Figure 6:**
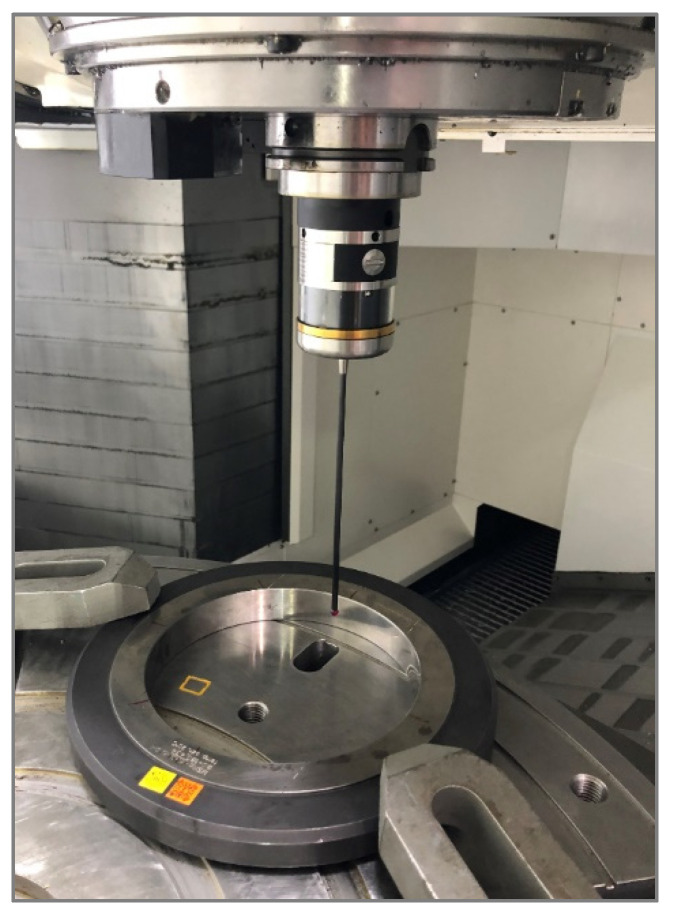
Test setup of ring gauge DIA. 180.4410 mm for initial R&R probing system performance analysis, repeatability, and reproducibility, T1 TTP.

The R&R method was used for the definition of the repeatability and reproducibility index value of the measuring system for the T1 probe, the most important probe in the system. The R&R evaluation has been performed for both MTs #2 and #3 according to the methodology as proposed in the article [28]. Initial test setup is presented in Figure 6.

The R&R measurement evaluation was performed based on two sequences as follows:

- Sequence 1, measurement of ring gauge DIA. 180.4410 mm, in reference to established working base. Measurement of DIA., *X* and *Y* true position and *Z* level surface. Each measurement separated by *X*, *Y*, *Z* axis moves from G28 reference point, *B* and *C* axis move ±15°. Data separation for *X* and *Y* axis results (according to MT coordinate system), single point measurement of *Z* level. Automatic mode.
- Sequence 2, measurement similar to Sequence 1, the same working base, with probe load and unload into the spindle from tool magazine before each measurement. Automatic mode.

R&R measurement evaluation has been simplified and adopted in reference to that presented in the article [28] from four to two last sequences due to the major impact of measuring system quality. The R&R test was performed under shop floor conditions (temp. 24–25 °C) and without special MT preparation in between regular production.

The R&R elaboration methodology is based on articles [28,29]:

- The repeatability index Equipment Variation (EV) is determined by the following formula:
  EV = R_AVE_ K_1_ (1)
  where K_1_ is a constant depending on the numbers of parts and repetitions. In this specific case, factor K_1_ = 0.8862 assumed based on two test repetitions. The R_AVE_ is the average value of measurement error for the main probe, T1.
- The reproducibility index Appraiser Variation (AV) refers to the ability of a process or test to be duplicated by other operators. The AV index is determined by the following formula [17,18]:
  (2) $$\mathrm{AV}=\sqrt{{(X}_{\mathrm{diff}}K_{2}-\frac{{\mathrm{EV}}^{2}}{\mathrm{nr}}}$$
- The discussed R&R is made in the automatic mode, without operator influence. In this specific case, factor K_2_ = 0.7071 is assumed based on two test repetitions treated as performed by two separate “operators”. The Xdiff is the spread of the means for individual sequences [17], variable n is number of test parts—in this case, n = 1 (master ring only)—and variable r is the number of repetitions—in this case, r = 32 for MT #2 and r = 22 for MT#3.
- The R&R expanded uncertainty expression is determined by the following formula [17,18]:
  (3) $$R\&R=\sqrt{{\mathrm{EV}}^{2}{+\mathrm{AV}}^{2}}$$
- In the case of complex R&R analysis, the percentage tolerances analysis was calculated in relation to manufacturing process requirements. The percentage tolerances analysis is calculated based on correlation [17,18]:
  (4) $$\%\mathrm{EV}=\frac{\mathrm{EV}}{T}100\%\;$$
  (5) $$\%\mathrm{AV}=\frac{\mathrm{AV}}{T}100\%\;$$
  (6) $$\%R\&R=\frac{R\&R}{T}100\%\;$$

Section 4.1 shows detailed results of the R&R evaluation of the T1 main probe in relation to both test cells: MT #2, MT #3.

### 3.3. Comparative Performance Evaluation Methodology

The subject of our research was to determine the value of mutual errors between various technological configurations of TTP in the in-machine workpiece measuring system. We aimed to determine the suitability of a specific configuration of TTP for the given technological process requirements. The goal of a test configuration is to determine the errors of a particular probe type in relation to the measurement of coordinate points (center of the diameter), the value of the reference diameter, single-point measurement, the distance to the *C*-axis rotation point of the table, and the distance of the *Z* axis from the G28 reference point.

The measurement sequence and scheme follow the MT configuration, machining part requirements, and probing usage in both turning and milling modes.

Due to the 5-axis machine tool’s tilting spindle head design, the measurement was performed in the vertical position (*B*0° head position) for the T1, T2, T9, T5, and T4 probes and the horizontal position (*B*90° head position) for T1, T2, T9, T5, and T6.

Chapter 6.7 of the ISO 230-10: 2016 standard [22,27] has become the basis for developing a dedicated methodology, test measurement schemes, as well as our own concept of MT inspection for artifact patterns in TTP systems. 

**Figure 7:**
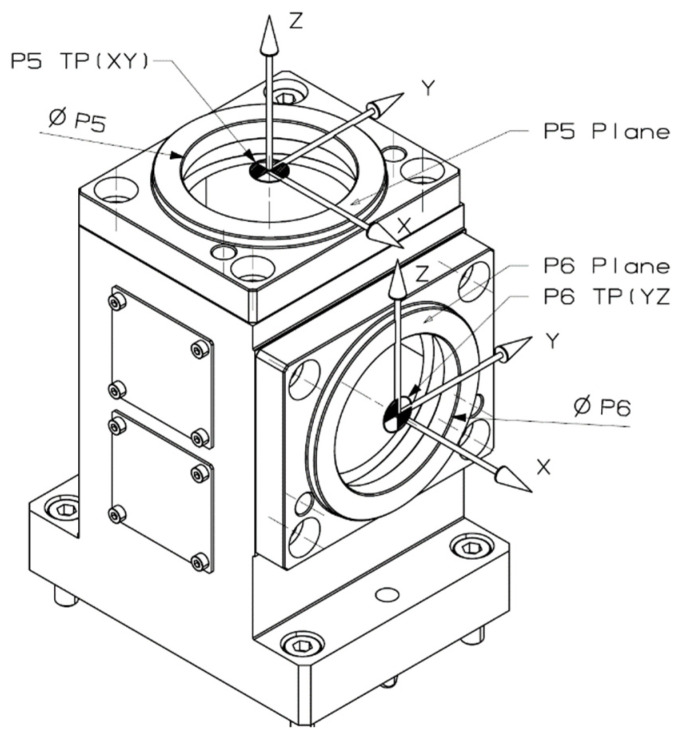
The artifact concept for the TTP system performance test—custom artifact for 5-axis MT with tilting head: *B*0° P5 position, G54.1P290 working base; P5 surface, plane A; P5 DIA., bore B; *B*90° P6 position, G54.1P291 working base; P6 surface, plane C; P5 DIA., bore D.

Figure 7 shows the characteristics of geometric test artifacts and positioning ideas, dedicated artifact shape concepts based on machining process requirements, and MT 5-axis tilting head configurations.

**Figure 8:**
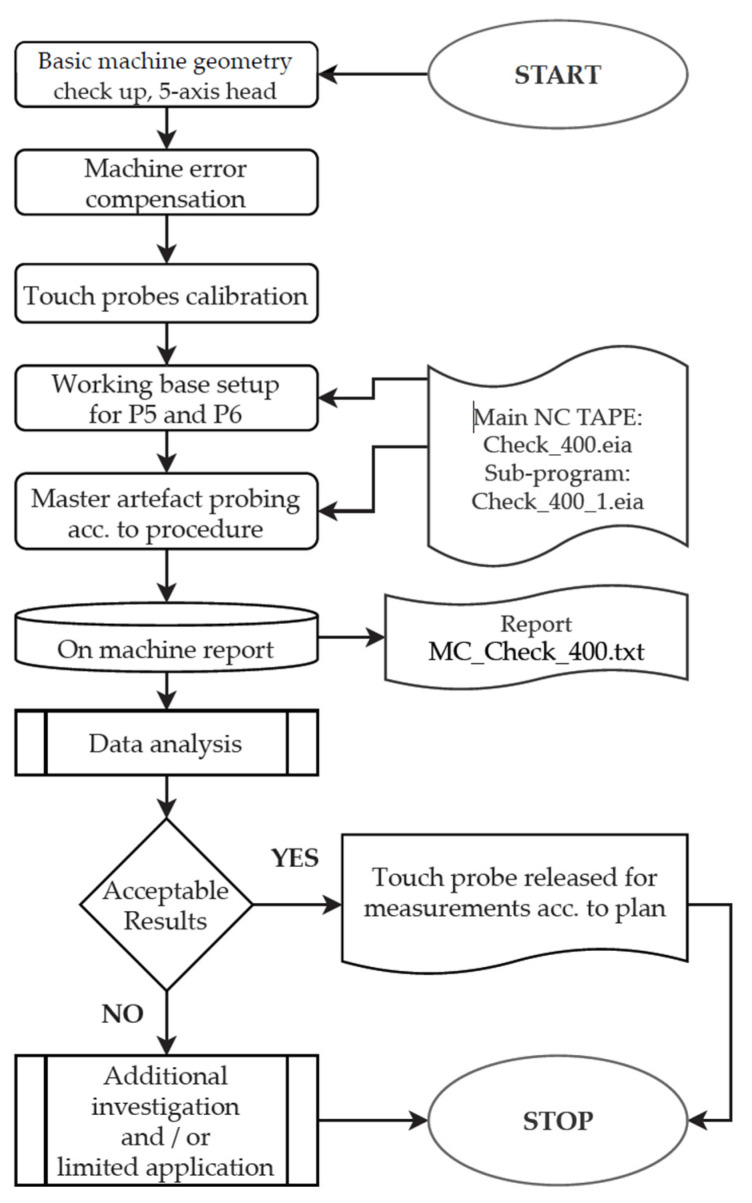
Flow chart of probing system performance proof.

**Figure 9:**
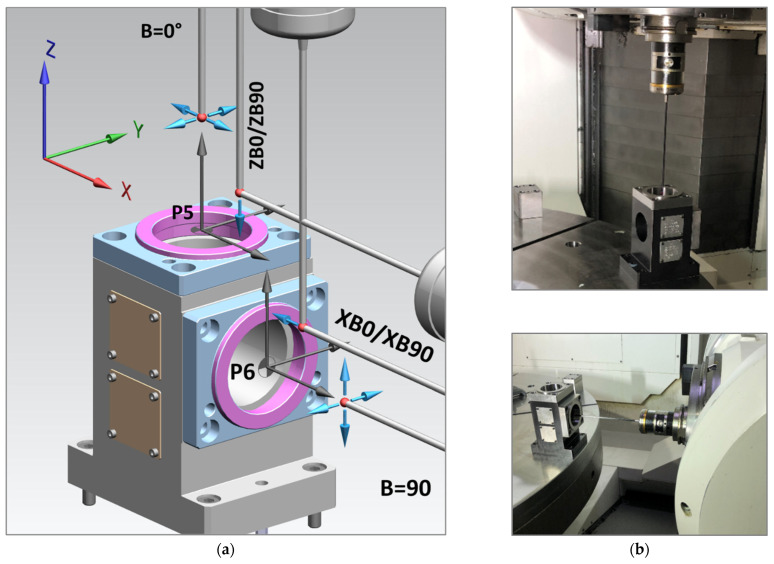
Visualization of artifact probing sequence procedure: (**a**) TTP stylus approach for each of the measurement areas and points; (**b**) MT measurement examples with T1 TTP configuration vertical *B*0° position and horizontal *B*90°, artifact built in special calibration fixture with periodic validation.

**Figure 10:**
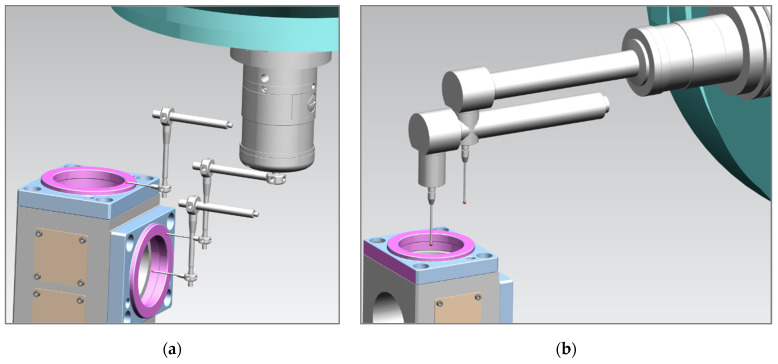
View of required B-axis tilting head position for measurement by special or custom probes: (**a**) T4 TTP position for P6/P5 area measurement with *B*0° head position; (**b**) T6 TTP position for P5 area measurement with *B*90° head position.

The idea behind our test methodology is shown in Figure 8, visualized in Figure 9 and Figure 10, and described below:

1. A basic machine geometry inspection is conducted according to ISO 10579-1 G12 [35], which assesses the 300 mm test bar to limit the direct impact of tilting head errors influencing the results.
2. Machine error compensation is conducted using the software Intelligent Mazacheck, Yamazaki Mazak Corporation [36].
3. Calibration of all TTPs based on dedicated artifacts—master ball ø25 mm and/or master ring ø60 mm, separate customized NC macro programs for each probe (automated calibration for special fixture), each probe has separate variables of calibration data.
4. Working base coordinates *XYZC* setup: G54.1 P290 for P5 master block with DIA. 59.990 mm to *B*0° head position and G54.1 P291 for P6 master block with DIA. 59.988 mm to *B*90° head (artifact built in special fixture with periodic validation).
5. Artifact measurement by sequences is shown in Figure 9 and Figure 10 with the measuring points positioned exactly the same for each probe. Custom NC tape, main program CHECK_400.eia with sub-program CHECK_400_1.eia.
6. Measurement data stored on #800-879 variables and recorded in MC_Check_400.TXT file.

Complete measurement is performed in auto mode without operator intervention. Measurements performed (for all probes) with the following parameters:

- P5 DIA. = 59.990 mm; 3 mm depth from plane P5, 4-point macro cycle;
- P5 plane Z measurement—point *X* = 37 mm from P5 center (*X*0 *Y*0);
- P6 DIA. = 59.998 mm; 3 mm depth from plane P6. 4-point macro cycle;
- P6 plane *X* measurement—point *Z* = 37 mm from P6 center (*Y*0 *Z*0);
- First touch feed = 1500 mm/min;
- Second touch feed = 100 mm/min.

The probing pattern in conjunction with the probe configuration is shown in Table 3. The probing pattern depends on the MT’s tilting head angular position (B-axis head) and stylus access. Test NC tape is used to measure macrocycles from the software Inspection Plus Special with NX CAM system support.

After performing the preliminary steps 1–4 and setup for the MT, the CHECK_400.eia NC program performed an automatic cycle according to the following general sequence:

1. T1 TTP measure DIA. P5 and true position of DIA. P5, *B*0° head position;
2. T1 TTP measure surface P5 by *Z*-axis movement and next P6 surface by *X*-axis movement, *B*0° head position;
3. T1 TTP measure DIA. P6 and true position of DIA. P6, *B*90° head position;
4. T1 TTP measure surface P6 by *X*-axis movement and next P5 surface by *Z*-axis movement, *B*90° head position;
5. T2 & T9 automatic change and performing the same sequence as T1;
6. T5 automatic change and sequence performed by (1) and (3);
7. T6 automatic change and sequence performed by (1), (2), and (4) items, *B*90°, Figure 10b;
8. T4 automatic change and sequence performed by (3) and (4) items *B*0°, Figure 10a.

Figure 10a,b show the T4 and T6 *B*-axis head demand position for measurement in reference to the probe shape configuration.

While measuring T1, T2, and T9 TTP, the *B*-axis pivot error is also calculated for two positions [37]:

- As a distance measurement error for the *Z*-axis of the *B*0°/90° position:
  #810 = #803 − #809(7)
- #803 results from P5 plane *Z*-axis point distance measurement with *B*0° head position and #809 results from P5 plane *Z* axis for the same point distance measurement with *B*90° head position, 4;
- As a distance measurement error for *X* axis of *B*0°/90° position:
  #811 =#804 − #808(8)
  #804 results from P6 plane *X*-axis point distance measurement with *B*0° head position and #808 results from P6 plane *X*-axis plane measurement with *B*90°.

9. Data write from NC #800 to #879 in to CHECK_400.txt report file.

The collected measurement data, based on the described diagram, are presented in detail in the next section of the article.

## 4. Results and Analysis

### 4.1. R&R Measurement System Evaluation of Reference T1 Probe

Table 4 presents elaborated results of R&R analysis of MT #2 and MT#3 with the dedicated T1 probe. 

The value of repeatability index Equipment Variation (EV) and the average value of measurement error (R_AVE_) of the main T1 probe are shown in Table 4, columns 7 and 8.

The reproducibility index Appraiser Variation (AV) and the spread of the means for individual sequences (Xdiff) are presented in Table 4, columns 6 and 9.

The R&R expanded uncertainty values are presented in Table 4, column 11.

In the case of complex R&R analysis, the percentage tolerances are calculated in relation to manufacturing process requirements. Table 5 shows the process tolerances and specific and tight requirements of parts machined on the analyzed MT #2 and #3.

The results of %EV, %AV, and %R&R are presented in Table 4, columns 11, 12, and 13.

The calculated results %EV, %AV, %R&R are acceptable according to the criteria presented in the article [18]; however, %EV, %R&R of DIA. are acceptable conditionally (values > 10% > 30%) for MT#2. The results of %EV, %AV, %R&R are affected by the variable shop floor conditions (lack of thermal stabilization on the shop floor) and ring gauge position in relation to *Y* axis > 50% of machine axis stroke. Based on the results as presented, the study proceeded to the second stage.

### 4.2. Comparative Performance Evaluation Analysis Study

Preliminary test data were collected according to the sequence and pattern shown in Figure 9a and Table 3, with the three straight probes T1, T2, and T9 on MT#3, to confirm the study concept.

Our main research consisted of five tests performed on MT#3 and six tests performed on MT#2, with dedicated whole probe sets for each machine. Table 6 shows an example of a set of results from one complete measurement with a full batch of probes according to the sequence and pattern shown in Figure 9a and Figure 10a,b and Table 3.

**Figure 11:**
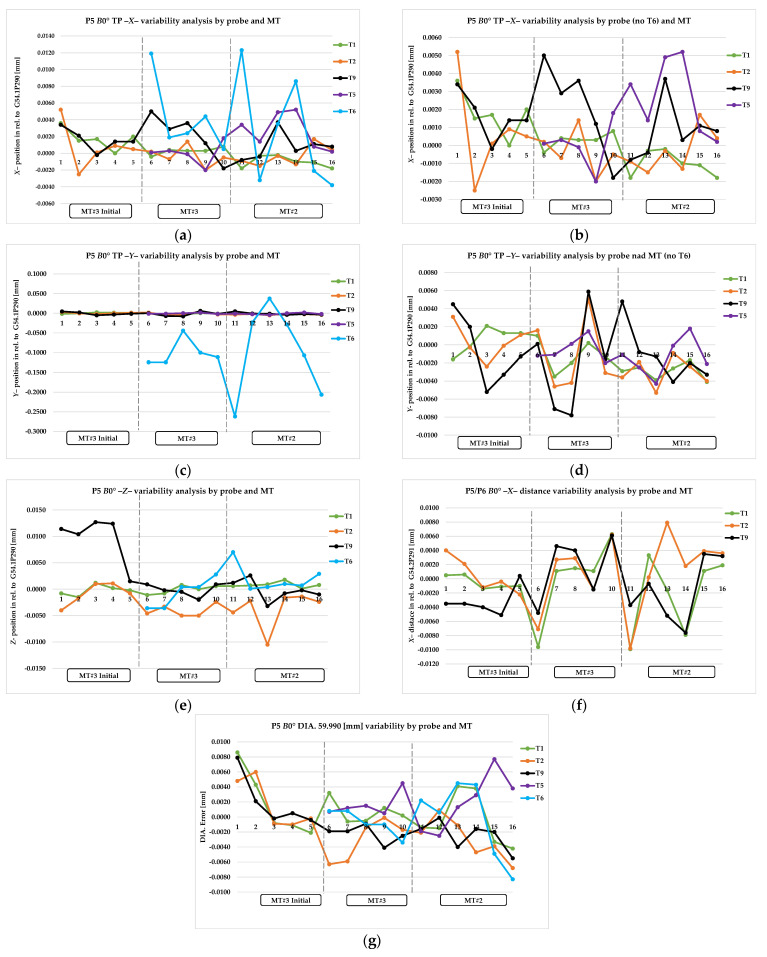
Analysis of P5 area measurement for the probes T1, T2, T9, T5 (*B*0° head), and T6 (*B*90° head): (**a**) variability of hole true position—*X* direction, all probes; (**b**) variability of hole true position—*X* direction, T6 probe error hidden; (**c**) variability of hole’s true position—*Y* direction, all probes; (**d**) variability of hole’s true position—*Y* direction, T6 probe error hidden; (**e**) variability of *Z*– direction distance; (**f**) variability of *X*– direction distance; (**g**) variability of master hole size.

**Figure 12:**
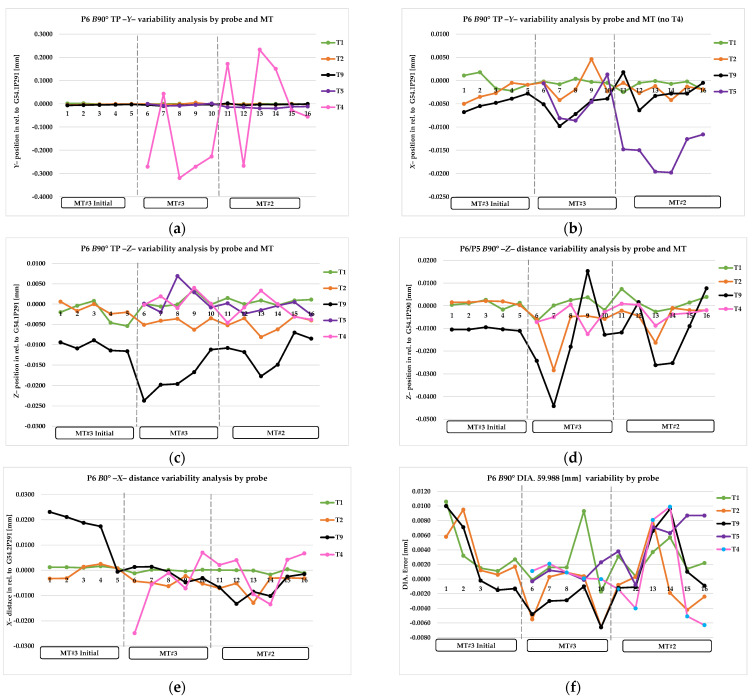
Analysis of P6 area measurement for probes T1, T2, T9, T5 (*B*90° head), and T4 (*B*0° head): (**a**) variability of hole true position—*Y* direction, all probes; (**b**) variability of hole’s true position—*Y* direction, T6 probe error hidden; (**c**) variability of hole’s true position—*Z* direction; (**d**) variability of *Z*—direction distance; (**e**) variability of *X*—direction distance; (**f**) variability of master hole size.

**Figure 13:**
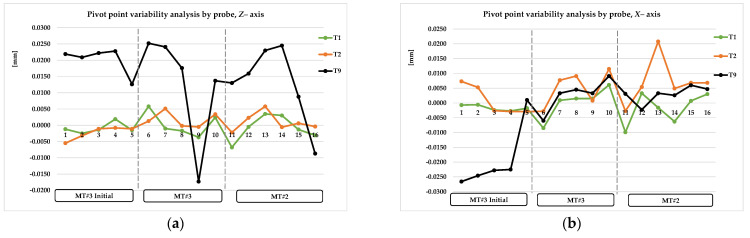
Pivot point measurement variability: (**a**) *Z*—axis direction, *B*0° → *B*90° head position; (**b**) *X*—axis direction, *B*0° → B90° head position.

TP *X*0 and TP *Y*0 refer to the error value of the measured true position of the diameter of the *X* and *Y* axis of P5 point, *B*0° head position. DIA. refers to the value of measured diameter. ZB0 and XB0 refer to the error value of the reference surface of pivot point calculation, *B*0° head position. TP Y90 and TPZ90 refer to the error value of the measured true position of the diameter of the *Y* and *Z* axis of P6 pint, *B*90° head position. XB90 and ZB90 refer to the error value of the reference surface of pivot point calculation, B90° head position. The *Z*error and *X*error refer to the pivot point error value, calculated based on Equations (7) and (8). The complete collected data are presented graphically in Figure 11a–g, Figure 12a–f, and Figure 13a,b and summarized in Table 7, Table 8 and Table 9.

Based on the prelimary test data, significant differences were recognized in the measurement results for the T9 probe in the *Z* axis for both the MT spindle head position *B*0° (P5 area) and *B*90° (P6 area). The results are presented in Figure 11, Figure 12 and Figure 13 in sequence #1–#3.

**Figure 14:**
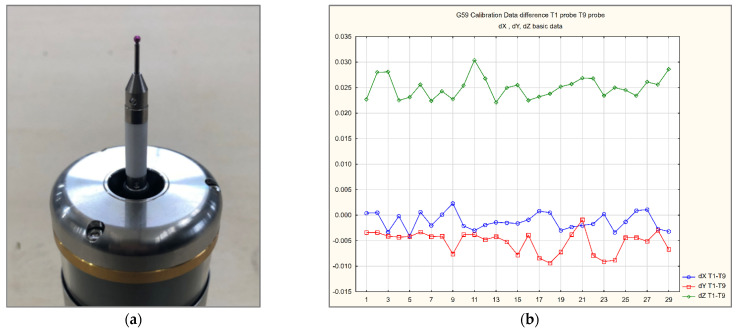
Preliminary stage error investigation features: (**a**) alternative T9 probe shape used for error investigation (preliminary stage); (**b**) run chart of error range between the probes T1 and T9; the *XYZ* coordinates of calibration working base determined automatically through a dedicated calibration cycle.

Two additional tests were performed to diagnose the source of the issue. During test #4, the T9 probe was replaced with an entirely new TTP set (holder, RMP600 probe kit, and stylus). The results were similar to the previous ones, which excluded the incorrect behavior of the previously installed probe. The next stage #5 of the search for the origin of error involved changing the configuration of the measuring stylus to an alternate one. We chose a stiffer type that used a ceramic extension L = 30 mm and shorter steel styli L = 20 mm with a ruby ball measuring ø2.0 mm. Figure 14a shows alternative configurations of the T9 probe shape. The results of #5 stage were again similar to previous data from measurements 1–4.

In order to eliminate the measurement error in the *Z* axis for the head position B0°, probe length compensation error was introduced at the T9 probe calibration stage (part machining process requirement—high correlation of results between T1 and T9) with the continuous monitoring of the probe’s calibration parameters (Figure 14b). The error source is further described in Section 4.3.

### 4.3. Analysis and Effects

In order to gain a clear interpretation of the results, a numerical analysis was conducted, and assumptions and observations were noted. The most important of these were as follows: Figure 11a–g and Table 7 show the results of probing variability for the P5 artifact area (B0° head position except T6); Figure 12a–f and Table 8 show probing variability for the P6 artifact area (B90° position except T4). 

The results of the T1, T2, and T5 probes were consistent with our assumptions for the dimensional conditions and true position condition requirements. Moreover, T5′s probe performance in the horizontal head position on MT#2 was much lower than MT#3; the range was −0.020 mm in the *Y*-axis true position (Figure 12b, Table 8 line 9–10). 

**Figure 15:**
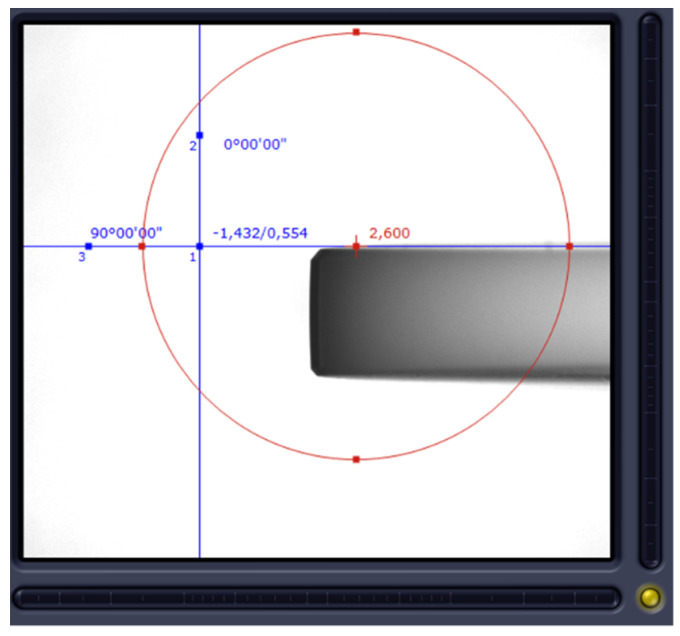
Example of T5 probe error investigation. Disc styli visual valuation on the tool pre-setter camera.

The T5 probe’s error source was analyzed extensively. The calibration data were compared between the T5 MT#3 probe and T5 MT#2 probe. The data comparison is presented in Table 10 (lines 3–5 and 7–9). No significant differences were recognized in relation to T5 MT#2 data. The probe complete set assembly was checked (Figure 15). No issue was preliminary recognized.

Later, during the periodic calibration of the T5 probe on MT#2, a significant error occurred.

The protective gate of the NC tape stopped the calibration operation in case of out-of-tolerance values of styli radius and disc axis offset (Table 10, line 11). Additionally, disc run-out could not be eliminated by alignment with the dial indicator.

**Figure 16:**
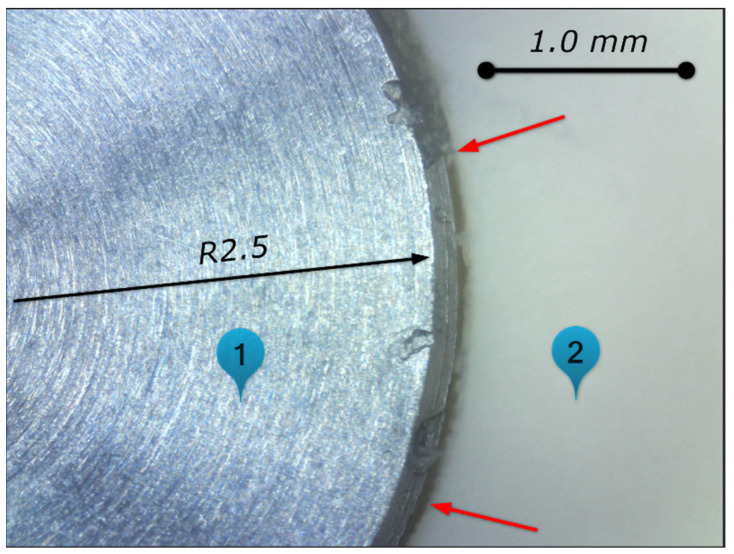
T5 MC#2 styli damage—lack of glue joint around sleeve and ceramic disc. 1—sleeve area, 2—ceramic disc area. 200× magnification.

Finally, the error root cause was recognized: the glue joint between the ceramic disc and stainless-steel sleeve was damaged. Figure 16 shows the damaged area.

The damaged parts were replaced, i.e., the styli and extension. Table 10, lines 13–15 shows current calibration data and Table 11 shows measurement verification after styli replacement. The measurement results are better; error values are more comparable for the T1 probe without significant error of the *Y* axis. The previous behavior of the T5 MT#2 probe could be affected by progressive styli damage.

The results referenced in Section 4.2. of R&R (Table 4) and data shown in the Table 7, Table 8 and Table 9 and 11 indicate probing performance less than ±10 µm of the T1, T2, and T5 probes dimensional requirements. This should be considered a valid result for mid-sized, long-term-use MTs under production conditions with temperature variation (Table 12). 

The T1, T2, and T5 probes can be used for all application tags as defined: setup, adaptive process, measurement of features, and MT error compensation. However, R&R of the main T1 probe in comparison to a similar probe configuration, as shown in the article [28], is significantly lower. The difference is caused by the process tolerance requirements, machine tool size, and 5-axis configuration (tilting head instead of swiveling table, rigid column).

The T4 and T6 probes demonstrate the significant influence of probe shape configuration compared with the MT configuration. The high value of the true position error for the *Y* axis depends on the repeatability of the spindle tool clamp’s angular position (Figure 11c and Figure 12a, Table 7 line 11–12, Table 8 line 11–12). The machines used in the test were equipped with HSK-A/T 100 shanks and spindles positioned at 1°. The mechanical mounting error resulting from the gap between the spindle key and keyway of the probe holder causes the probe’s lack of repeatability in the spindle mounting; this directly affects the deviation of the stylus tip from its angular position in relation to the *Y* axis. The incorrect angular position also affects the calibration compliance of the T4 probe length in the *X* axis (Figure 12e, Table 8 line 11–12), which significantly affects the dimensional accuracy of hard-to-reach diameters machined in the turning process. MT automatic tool change systems in conjunction with T4/T6 probe configurations do not allow the usage of additional elements for angular probes in the fixed spindle position to eliminate mounting errors (fixed spindle position prohibited by the spindle probe rotation of automatic tool change). The diameter and *Z*-level measurement results are consistent with our assumptions for the dimensional condition requirements. T4 and T6 probes can be used for limited applications where the angular position of the probe does not affect the measurement result, including setup, adaptive process, and features measurement. Due to performance tolerance, these results have been conditionally accepted. In order to eliminate the behavioral influence of the T4 probe on diameters (turning mode) with a tolerance lower than ±0.025 mm, a new probe called T10 was designed (implementation stage) for hard-to-reach diameters and grooves with research experience application of T4 probe use.

The results for the T9 probe, in the *B*0° head, vertical position, are consistent and correlate with the results of the T1, T2, and T5 probes in reference to the true position error (*XY* plane) and *X*, and currently the *Z*-axis distance too (Figure 11a–g, Table 7 line 7–8). The results for the T9 probe, in the *B*90° head, horizontal position, are not consistent with the *Z*-axis true position and *Z-* and *X*-axis surface measurement (Figure 12c,d, Table 8 line 7–8). The *Z-* and *X*-axis error (*B*0°–*B*90°) was obtained by modifying the probe length’s calibration cycle. However, variability of the *Z*-axis distance (*B*90° position) is still higher than that for the T1 or T2 probe (Figure 12d, Table 8 line 7–8). The probe length’s correction level is currently between 0.010 and 0.020 mm. In relation to the horizontal position of the *B*90° head, the hole’s true position and the height along the *Z* axis are still affected by an error of 0.013–0.025 mm and are not stable (Figure 12c, Table 8 line 7–8; Figure 13a, Table 9 line 8–9). 

The error source investigation, initially, was focused on the probe configuration.

Complementary tests conducted with an additional new probe and alternate configuration with a ceramic extension and shorter stylus tip did not improve the results. Table 13 shows the measurement results for three configurations of T9 probes used in the preliminary research stage. There are no significant differences between the obtained results.

The next step of investigation was the calibration process of the T9 probe. The T1, T2, and T9 probes used the same, automated macro program of probe calibration. During the calibration cycle, the *Z*-level of the master ball is set automatically by macro O9600 [33] (*B*90° position). However, the Z position of the calibration artifact is shifted for T9 within 0.020–0.030 mm in relation to T1 and T2. Figure 14b shows this phenomenon as a run chart with monitoring of calibration data correlations between the main probes T1 and T9. The similarity of the error value between MT#2 and #3 confirms that the error source is not connected directly with the MT’s behavior, but is more related to the probe behavior, with short styli in the horizontal position of measurement. The calibration working base shift converges with the hole artifact’s true position error measurement (Figure 12c–e).

The planned improvement of T9 covers the preparation of a separate T9 probe calibration cycle to remove the systematic measurement error.

Table 14 shows the TTP performance summary of the analyzed probing system. The data are consistent with Table 15 [13], which shows the cumulative probing error budget for small and medium sizes of MTs. Due to the principles of probing system operation, MT errors are directly impacted by the probing results. The geometric and position accuracy of the five-axis head in our study are also particularly important. Trial #5 for MT#3 and trials #10–11 for MT#2 were performed after periodic MT geometry inspection and correction. The error range for all measuring points is tighter within 0.005 mm. This observation is visible in the charts for measuring items 10 (MT#3, test #5) and 15/16 (MT#2, test #5 and 6). We also compared the behaviors of two MTs (same type). There were no significant differences in the results, except for the increased range of diameters measured in trial #6 on MC#2 MT (T5′s horizontal *Y*-axis error cancelled).

Our methodology for assessing the suitability of probes can also be used as a tool for diagnosing machine tool errors; this mainly applies to the probes T1, T2, and currently T5. In the case of significant error values in the true position between probes, detected direction, and deviation tendency, it may be possible to recognize deviations in machine tool geometry as quick, simultaneous assessment tools for MT and TTP.

## 5. Summary

In this article, we describe a dedicated methodology for comparative performance evaluations of multiconfiguration touch-trigger probes. This method can be used for standard, straight, and special probes of non-standard shape.

The presented solution has been implemented as a dedicated tool for probing system performance analysis in shop floor environments. The advantage of these methods is that each probe error can be identified and analyzed to determine the root cause. A clear disadvantage of this method is that the shop floor environment affects the results, especially temperature. However, the purpose was to develop a method for determining the probing system’s error value and error range in relative production conditions.

Based on the measurement results and their analysis, the following observations were made:

- The presented method is not intended to eliminate the R&R methodology of probing system evaluation; however, it can support statistical methods with a quick and direct shop floor diagnostic tool for probing performance analysis;
- TTP measurement results were affected by the MT errors and temperature variations; TTP shape configuration and styli shape had an essential influence on the measurement results in reference to special probes T4 and T6;
- T9 *B*90° errors required further analysis of the calibration strategy for the use of the T9 probe throughout its full range of applications;
- A suggested improvement of this work is the implementation of an additional test stage with a three-points measurement strategy for diameter measurement—the three-points approach is also used for measurements where a four-points strategy is not suitable, e.g., limited access;
- The methodology can be implemented in the periodic validation schedule of MT probing systems to collect more data with a specific timeline for future prediction of system conditions (e.g., T5 MT# probe performance loss);
- The functionality and flexibility of dedicated artifacts can be improved with an additional reference plate on the back of the support body.

The ISO230-10 standard [27] supports the validation process of the integrated measuring system of the machine. However, for special, non-standard applications, a single solution is required to initially determine the measuring system’s suitability.

The universality of the presented method is generally independent of the MT size and machine kinematic layout (tilting head or tilting table). The method and equipment can be easily adapted to dedicated probing system requirements.

## Figures and Tables

**Table 1:** General scope of the 5-axis multitasking machine tool used in our study—MT specification outline.

Test Cell—Machine Tool #2 and #3			
Capability	Max. workpiece size	ø2050 × 1600	[mm]
	Max. pallet load	3750	[kg]
Axis stroke	*X*	2315	[mm]
	*Y*	1600	[mm]
	*Z*	1345	[mm]
	*B*	−30 to +120	[°]
	*C* axis	360	[°]
Spindle	Shank type	HSK A/T100	
	Index for probing	1	[°]
CNC controller	Mazatrol	SmoothX	
Probing software	Renishaw	Inspection Plus Special	

**Table 2:** Touch-trigger probe configuration included in an advanced on-machine measurement system [34]. Application tags: S, setup; A, process adaptive; C, measurement; M, machine tool checkup and calibration.

No.	Type	Stylus Shape	Tip Shape	Tip Size [mm]	L1 Length [mm]	L2 Length [mm]	Sense Direction	Unidirectional Repeatability [mm]	Stylus Type	Application Area
T1	RMP600	Straight	Ball	6	340	-	*±X, ±Y, +Z*	±0.00035	Carbon fiber	S, A, C, M
T2	RMP600	Straight	Ball	2	288	-	*±X, ±Y, +Z*		Ceramic Extension + Tungsten steel	S, A, C
T4A	RMP600	Z-shape	Ball	2	211	99.5	*±X, ±Y, ±Z*		Custom, Tungsten steel	S, A, C
T4B						−99.5				
T9	RMP600	Straight	Ball	2	188	-	*±X, ±Y, +Z*		Tungsten steel	S, A, C
T5	RMP60M + LP2	Straight	Disc	28	515	-	*±X, ±Y*	±0.001	Custom, Ceramic	C, M
T6	RMP60M + LP2	L-shape	Ball	3	315	−107.5	*±X, ±Y, +Z*		Tungsten steel	A, C

**Table 3:** Probing pattern for all probe configurations used for the test.

Probe No.		T1	T2	T9	T4A	T5	T6
Head Position		*B*0°/*B*90°	*B*0°/*B*90°	*B*0°/*B*90°	*B*0°	*B*0°/*B*90°	*B*90°
P5	DIA. ^1^	•	•	•		•	•
	TP *XY* ^2^	•	•	•		•	•
	*Z*0 ^3^	•	•	•	•		•
P6	*X*0 ^4^	•	•	•			
P6	DIA.	•	•	•	•	•	
	TP *YZ* ^5^	•	•	•	•	•	
	*X*90 ^6^	•	•	•	•		
P5	*Z*90 ^7^	•	•	•			

^1^ Diameters of maser ring. ^2^ True position of diameter P5, error separated of *X* and *Y* axis. ^3^ Reference surface P5 of Z value measurement. ^4^ Reference surface P6 of X value measurement. ^5^ True position of diameter P6, error separated of *X* and *Y* axis. ^6^ Reference surface P6 of X value measurement. ^7^ Reference surface P5 of Z value measurement.

**Table 4:** The R&R analysis results of MT#2 and #3 measurement system quality, repeatability, and reproducibility values.

MC No.	Probe	Feature	Min.	Max.	Xdiff	R_AVE_	EV	AV	R&R	%EV	%AV	%R&R
		Value	[µm]	[µm]	[µm]	[µm]	[µm]	[µm]	[µm]	%	%	%
R&R analysis MT#2	T1	DIA. Err.	1.0	6.2	0.2	4.4	3.9	0.0	3.86	15.4%	0.0%	15.4%
		*X*	−0.8	1.2	0.3	0.1	0.1	0.2	0.24	0.4%	1.1%	1.2%
		*Y*	−1.5	2.2	2.1	0.6	0.5	1.5	1.59	2.1%	7.5%	7.9%
		TP	0.4	4.4	1.9	2.5	2.2	1.2	2.57	9.0%	3.3%	6.8%
		*Z*	−0.8	1.9	0.9	0.7	0.6	0.7	0.92	2.6%	2.6%	3.7%
R&R analysis MT#3	T1	DIA. Err.	1.6	3.5	0.8	2.4	2.1	0.3	2.16	8.6%	1.1%	8.7%
		*X*	−0.9	2.1	1.2	0.3	0.3	0.8	0.89	1.1%	4.2%	4.4%
		*Y*	−0.6	3.2	2.3	1.2	1.1	1.6	1.94	4.4%	8.0%	9.7%
		TP	0.8	4.3	2.3	2.4	2.2	1.6	2.66	8.6%	4.1%	7.0%
		*Z*	−2.1	1.4	−0.3	−0.5	−0.4	0.2	0.48	−1.7%	0.9%	1.9%

**Table 5:** The manufacturing tolerances of machining process requirements.

	Characteristic	T	Unit
Diameter error	DIA.	25	[µm]
*X*-axis position error	*X*	20	[µm]
*Y*-axis position error	*Y*	20	[µm]
True position of diameter axis	TP	38	[µm]
*Z* level of base surface position	*Z*	25	[µm]

**Table 6:** Example of collected test data from one set of measurements, all types of probes.

	P5 *B*0°					P6 *B*90°					Pivot	
Probe No.	TP *X*0	TP *Y*0	DIA.	ZB0	XB0	TP *Y*90	TP *Z*90	DIA.	XB90	ZB90	*Z*error	*X*error
T1	−0.0018	−0.0029	59.9886	0.0006	−0.0099	−0.0025	0.0015	59.9911	0.0001	0.0074	−0.0068	−0.0099
T2	−0.0009	−0.0036	59.9879	−0.0044	−0.0098	−0.0005	−0.0052	59.9872	−0.0071	−0.0022	−0.0022	−0.0027
T9	−0.0008	0.0048	59.9884	0.0012	−0.0037	0.0018	−0.0108	59.9868	−0.0068	−0.0118	0.013	0.0031
T5	0.0034	−0.0011	59.9881	–	–	−0.0148	0.0002	59.9918	–	–	–	–
T6	0.0123	−0.2621	59.9922	0.0070	–	–	–	–	–	–	–	–
T4A	–	–	–	0.0009	–	0.1716	−0.0046	59.9866	0.0021	–	–	–
	MT No.	#2	Test No.	#6		Date	14 July 2020	Time	101908		Temp. [°]	26.5

**Table 7:** Measurement dispersion for P5 area *B*0° head positions for the probes T1, T2, T9, T5, and *B*90° head position for T6. Results in [mm].

	TP *X*0 Error		TP *Y*0 Error		DIA. Error		ZB0 Error		XB0 Error	
P5 Area	MIN.	MAX.	MIN.	MAX.	MIN.	MAX.	MIN.	MAX.	MIN.	MAX.
T1 MT#3	−0.0004	0.0036	−0.0035	0.0021	−0.0021	0.0086	−0.0015	0.0012	−0.0096	0.0063
T1 MT#2	−0.0018	−0.0002	−0.0041	−0.0017	−0.0042	0.0041	0.0001	0.0018	−0.0099	0.0033
T2 MT#3	−0.0025	0.0052	−0.0046	0.0051	−0.0063	0.0060	−0.0050	0.0011	−0.0071	0.0062
T2 MT#2	−0.0015	0.0017	−0.0053	−0.0009	−0.0068	0.0009	−0.0105	−0.0014	−0.0098	0.0079
T9 MT#3	−0.0018	0.0050	−0.0078	0.0059	−0.0041	0.0079	−0.0020	0.0127	−0.0051	0.0061
T9 MT#2	−0.0008	0.0037	−0.0041	0.0048	−0.0055	−0.0001	−0.0032	0.0026	−0.0076	0.0035
T5 MT#3	−0.0020	0.0018	−0.0020	0.0015	0.0005	0.0045	-	-	-	-
T5 MT#2	0.0002	0.0052	−0.0043	0.0018	−0.0025	0.0077	-	-	-	-
T6 MT#3	0.0005	0.0119	−0.1243	−0.0442	−0.0034	0.0008	−0.0036	0.0028	-	-
T6 MT#3	−0.0038	0.0123	−0.2621	0.0373	−0.0083	0.0045	0.0001	0.0070	-	-

**Table 8:** Measurement dispersion for P6 area, *B*90° head positions for the probes T1, T2, T9, T5, and *B*0° head position for T4. Results in [mm].

	TP *Y*90 Error		TP *Z*90 Error		DIA. Error		ZB90 Error		XB90 Error	
P6 Area	MIN.	MAX.	MIN.	MAX.	MIN.	MAX.	MIN.	MAX.	MIN.	MAX.
T1 MT#3	−0.0022	0.0018	−0.0054	0.0036	−0.0017	0.0106	−0.0069	0.0037	−0.0012	0.0037
T1 MT#2	−0.0025	−0.0001	−0.0003	0.0015	0.0004	0.0057	−0.0026	0.0074	−0.0017	0.0074
T2 MT#3	−0.0050	0.0046	−0.0063	0.0006	−0.0066	0.0095	−0.0284	0.0021	−0.0063	0.0025
T2 MT#2	−0.0042	−0.0005	−0.0081	−0.0030	−0.0042	0.0081	−0.0163	−0.0010	0.0025	0.0088
T9 MT#3	−0.0098	−0.0028	−0.0237	−0.0089	−0.0066	0.0100	−0.0442	0.0153	−0.0048	0.0231
T9 MT#2	−0.0064	0.0018	−0.0177	−0.0070	−0.0012	0.0096	−0.0262	0.0077	−0.0133	−0.0015
T5 MT#3	−0.0086	0.0013	−0.0020	0.0069	−0.0003	0.0023	-	-	-	-
T5 MT#2	−0.0198	−0.0116	−0.0026	0.0005	−0.0008	0.0087	-	-	-	-
T4 MT#3	−0.3193	0.0433	−0.0010	0.0040	0.0000	0.0021	−0.0125	0.0005	−0.0249	0.0070
T4 MT#2	−0.2670	0.2329	−0.0046	0.0033	−0.0063	0.0099	−0.0088	0.0009	−0.0135	0.0067

**Table 9:** Summary of results for *Z*- and *X*-axes pivot point error analysis for the probes T1, T2, and T9 at B0°/B90° head position. Results in [mm].

Head Pivot Error				
	*Z* Error		*X* Error	
Pivot	MIN.	MAX.	MIN.	MAX.
T1 MT#3	−0.0037	0.0058	−0.0085	0.0061
T1 MT#2	−0.0068	0.0035	−0.0099	0.0033
T2 MT#3	−0.0055	0.0051	−0.0029	0.0115
T2 MT#2	−0.0022	0.0058	−0.0027	0.0208
T9 MT#3	−0.0173	0.0252	−0.0060	0.0091
T9 MT#2	−0.0087	0.0245	−0.0023	0.0060

**Table 10:** Comparison of probing data of T5#3 and T5#2 probe. Key data shown. Results in [mm].

		T5 Probe. Disc DIA.		T5 Probe Disc Offset		Annotation
MT No.		R1.1 ^1^	R1.2 ^1^	Off1.1 ^2^	Off1.2 ^2^	
#3	min.	13.9440	13.9391	−0.0085	0.0027	
	max.	13.9481	13.9423	0.0021	0.0130	
	R ^3^	0.0041	0.0032	0.0106	0.0103	
	Q-ty ^4^	61				
#2	min.	13.9201	13.9223	−0.0235	−0.0068	
	max.	13.9245	13.9274	−0.0098	0.0047	
	R	0.0044	0.0051	0.0137	0.0115	
	Q-ty	101				
#2		13.8886	13.9232	−0.0653	0	calibration error
		out of tol. ^5^		out of tol.		
#2	min.	13.9212	13.924	−0.0037	−0.0015	new styliand extension
	max.	13.9266	13.9302	0.0171	0.0105	
	R	0.0054	0.0062	0.0208	0.0120	
	Q-ty data	78				

^1^ Disc radius of *X* and *Y* axis, ^2^ Disc axis misalignment in relation to the spindle axis,^3^ Range of results, ^4^ Quantity of calibration data in comparison, ^5^ CNC alarm based on built in NC tape limits.

**Table 11:** T5 MC#2 probe results dispersion, and comparison to T1 probe, new styli and extension.

**B0°**	**TP X0 Error**		**X Diff. ^1^**	**TP Y0 Error**		**Y Diff. ^1^**	**DIA. Result**		**DIA. Diff. ^1^**
**P5 Area**	**T1**	**T5**	**T5 to T1**	**T1**	**T5**	**T5 to T1**	**T1**	**T5**	**T5 to T1**
Test 1	0.0005	−0.0006	−0.0011	0.0013	0.0010	−0.0003	59.9862	59.9920	0.0058
Test 2	0.0009	−0.0017	−0.0026	0.0004	0.0020	0.0016	59.9869	59.9925	0.0056
Test 3	0.0011	−0.0006	−0.0017	0.0008	0.0004	−0.0004	59.9897	59.9919	0.0022
**B90°**	**TP Y90 Error**		**Y Diff. ^1^**	**TP Z90 Error**		**Z Diff. ^1^**	**DIA. Result**		**Dia. Diff. ^1^**
**P6 area**	**T1**	**T5**	**T5 to T1**	**T1**	**T5**	**T5 to T1**	**T1**	**T5**	**T5 to T1**
Test 1	−0.0012	−0.0020	−0.0008	−0.0020	0.0039	0.0059	59.9867	59.9904	0.0037
Test 2	−0.0010	−0.0027	−0.0017	−0.0021	0.0071	0.0092	59.9874	59.9910	0.0036
Test 3	0.0012	−0.0028	−0.0040	0.0016	−0.0017	−0.0033	59.9920	59.9924	0.0004

^1.^ Result difference T5 to T1.

**Table 12:** Temperature variation of shop floor area in relation to test number.

	Shop Floor Temperature during Tests [°C]															
Test No.	1	2	3	4	5	6	7	8	9	10	11	12	13	14	15	16
MT#3	24	24	24	25	25	24	27.5	27.5	26.5	29						
MT#2											26.5	27	25.5	25	28	28

**Table 13:** Comparison of probing data results with three sets of the T9 probe. Results in [mm].

		P5 B0°					P6 B90					Pivot	
Probe Config.	Note	TP X0	TP Y0	DIA.	ZB0	XB0	TP Y90	TP Z90	DIA.	XB90	ZB90	Z Error	X Error
T9	Basic ^1^	0.0021	0.002	59.9921	0.0104	−0.0035	−0.0055	−0.0109	59.9951	0.0211	−0.0105	0.0209	−0.0246
T9	New ^2^	−0.0002	−0.0052	59.9898	0.0127	−0.004	−0.0048	−0.0089	59.9878	0.0188	−0.0095	0.0222	−0.0228
T9	Alt. ^3^	0.0014	−0.0033	59.9905	0.0124	−0.0051	−0.0039	−0.0114	59.9865	0.0174	−0.0104	0.0228	−0.0225

^1^ Basic configuration of probe, regular operational use, ^2^ new set of basic probe, ^3^ alternative styli shape as shown in Figure 14a.

**Table 14:** Touch-trigger probe performance of analyzed probing system.

Probe Type	Probe No.	Attribute	Significance [µm]				Main Error Source
-			0–10	10–20	20–30	>30	
Straight	T1	Dimensional accuracy	•				MT accuracy (geometry and kinematics), temp. impact
		True position accuracy	•				
	T2	Dimensional accuracy		•			MT accuracy (geometry and kinematics), temp. Impact
		True position accuracy	•				
	T9	Dimensional accuracy		•			MT accuracy (geometry and kinematics), temp. impact, probe configuration, measurement position
		True position accuracy	• (vertical)		• (horizontal)		
	T5	Dimensional accuracy	•				MT accuracy (geometry and kinematics), temp. impact, styli impact (MT#2) ^1^
		True position accuracy		•			
L-type	T6	Dimensional accuracy		•			Probe configuration, MT spindle tool load reproducibility in relation tool angular orient
		True position accuracy				•	
Z-type	T4	Dimensional accuracy			•		Probe configuration, MT spindle tool load reproducibility in relation tool angular orient
		True position accuracy				•	

^1^ In relation to T5 MT#2 probe configuration.

**Table 15:** General error budget of part probing system for medium-sized MTs [13].

Error Source			Significance [µm]		
			0–10	10–20	50–100
Accuracy	MT geometry		•		
	TTP		•		
Repeatability	MT		•		
	Temperature effect				•
	Other effects			•	
Resolution	MT		•		
	TTP		•		

## Data Availability

Data are contained within the article.
